# Male Deep-Sea Shrimps *Aristeus antennatus* at Fishing Grounds: Growth and First Evaluation of Recruitment by Multilocus Genotyping

**DOI:** 10.3390/life11020116

**Published:** 2021-02-04

**Authors:** Alba Abras, Jose-Luis García-Marín, Sandra Heras, Manuel Vera, Melania Agulló, Laia Planella, María Inés Roldán

**Affiliations:** 1 Laboratori d’Ictiologia Genètica, Campus Montilivi, Universitat de Girona, 17003 Girona, Spain; alba.abras@udg.edu (A.A.); sandra.heras@udg.edu (S.H.); melania.agullo@udg.edu (M.A.); laia.planella@udg.edu (L.P.); marina.roldan@udg.edu (M.I.R.); 2 Departamento de Zooloxía, Xenética e Antropoloxía Física, Campus Lugo, Universidade de Santiago de Compostela, 27002 Lugo, Spain; manuel.vera@usc.es

**Keywords:** *Aristeus antennatus*, microsatellite loci, male recruitment, fishing grounds, deep-sea species

## Abstract

The population biology of the deep-sea shrimp *Aristeus antennatus*, as with other exploited demersal species, is usually studied using data from fishery statistics. Such statistical analyses have shown female-biased sex ratios during the spawning season in this species. Because the abundance of males increases at greater depths that are not exploited by fisheries (virgin grounds), knowledge on their recruitment is limited. Here, the growth and recruitment of *A. antennatus* males at fishing grounds was evaluated. This was achieved by integrating information on previously identified breeding behaviours and by tracing the young-of-year cohort through genotyping at 10 microsatellite loci. Using a codend and a codend cover with distinct meshed windows, four groups of males were collected in winter and in a subsequent spawning summer season. Summer collections were mostly composed of pre-adult males, reaching sizes that are to be expected from the growth of winter juveniles; however, many specimens also originated from nearby grounds. This result indicates the horizontal dispersal of male juveniles via intermediate and deep oceanographic currents. Such dispersal complements passive larval dispersal in surface waters, and contributes to the weak genetic divergence among regional fishing grounds. These features could be shared by other deep-sea crustacean and fish species, and should be considered for the sustainable exploitation of demersal fisheries.

## 1. Introduction

Demersal fisheries target many species of fish and crustaceans. While not representing the largest proportion of landings in Mediterranean countries, demersal species are highly sought after by fishermen, due to their high commercial value [1]. For instance, in the western Mediterranean, demersal stocks are subjected to high capture rates that are often above sustainable levels [2]. The blue and red shrimp, *Aristeus antennatus* (Risso, 1816) (Crustacea, Decapoda), is a typical target fish species of the demersal fisheries of northern Spain [3,4]. The commercial fishing of this species takes place by bottom trawling in submarine canyons, and near shallower waters, at depths of between 400 and 800 m [5]. However, in the western Mediterranean, this species is widely and heterogeneously distributed in the water column at depths ranging from 80 to 3300 m [6]. Peaks in abundance occur between 600 and 1000 m depth and then noticeably decline below 1500 m in depth [7]. Mature females are highly abundant at depths of less than 1000 m, whereas males and juveniles are more abundant in deeper layers (below the 1000 m) [7,8]. During the spawning season, from late spring to summer, aggregations of adult males and females occur at depths between 400 and 900 m [9]. Nevertheless, the genetic identification of spermatophores indicates that many mating males during the spawning season are not captured with females at these depths [10]. Males are more abundant at greater depths, where currently deep-sea fisheries are prohibited (below 1000 m; virgin areas) [7,11]. Consequently, as for other demersal exploited species, knowledge remains limited in terms of the growth and recruitment (either for reproduction or fishing) of male *A. antennatus*, as research on the population biology is primarily focused on specimens captured in fishing grounds [9,12,13,14,15]. 

The reproduction of *A. antennatus* was first described by Demestre and Fortuño in the fishing grounds of the western Mediterranean [16], with similar results being reported in other Mediterranean regions [13,17]. In brief, this species is sexually dimorphic, with an external mating system in which a sperm mass (spermatophore) attaches to the female’s open thelycum via the petasma of the male. Males are considered adults when they have fused hemipetasmas, and the rostrum measures less than 12 mm, otherwise they are classified as juveniles [16,18]. The carapace length (CL) of females has a wider range (CL = 18–68 mm) compared to males (CL = 18–36 mm). Females also have slightly greater longevity compared to males, reaching 5 and 4 years old, respectively [5]. The main reproductive period in the Catalan Sea, Spain (north-west Mediterranean) is highly seasonal, extending from late spring to summer, and peaking during summer [12]. A similar mating pattern was documented in the Balearic Islands (western Mediterranean) [19]. In comparison, longer periods were reported in the eastern Mediterranean, lasting almost seven months around Greece [17]. During the spawning season, the larvae of *A. antennatus* appear in surface waters (0.5 to 1 m depth) at areas where mature adults are fished at deeper layers [20]. After hatching on the bottom substrate, the first larval stages perform an ontogenic migration through the water column to the surface to optimize feeding and enhance development of the late larval stages [21]. Surface currents disperse these protozoea and zoea larval stages [22]. Finally, the decapodid stage organisms exhibit a downward vertical migration to deep waters, where they settle [7,23]. Small juveniles of 6–7 mm CL have been detected at 1300–1500 m depths after the spawning season [24]. A few months later, during winter, deep-sea *A. antennatus* juveniles recruit to wider depth ranges below 1000 m in the Catalan, Balearic, and Ionian Seas [11,24]. An abundance of juveniles of up to 15 mm CL has been reported at 2800 m depth [8]. In the north-western Mediterranean, juvenile numbers peak at the end of winter, in March [25,26]. Similar results were obtained in the eastern Mediterranean Sea, with juvenile shrimps mainly being reported from January to April [27].

The present study investigated the growth and recruitment of the juvenile and adult male *A. antennatus*, after the spawning season. This was accomplished by using multilocus genotype data to trace the young-of-year male cohort in the fishing ground of Palamós (Spain) as a case study, because of the available description of the breeding behaviour of the species during the spawning season in this area reported by Planella et al. [10]. This study aims to fill the knowledge gaps related to the growth and recruitment of *A. antennatus* males to fishing grounds and spawning aggregations. The present work is expected to provide baseline of information facilitating the sustainable exploitation of this marine resource, with potential application to other demersal species.

## 2. Materials and Methods

### 2.1. Sampling and Biological Material

**Figure 1:**
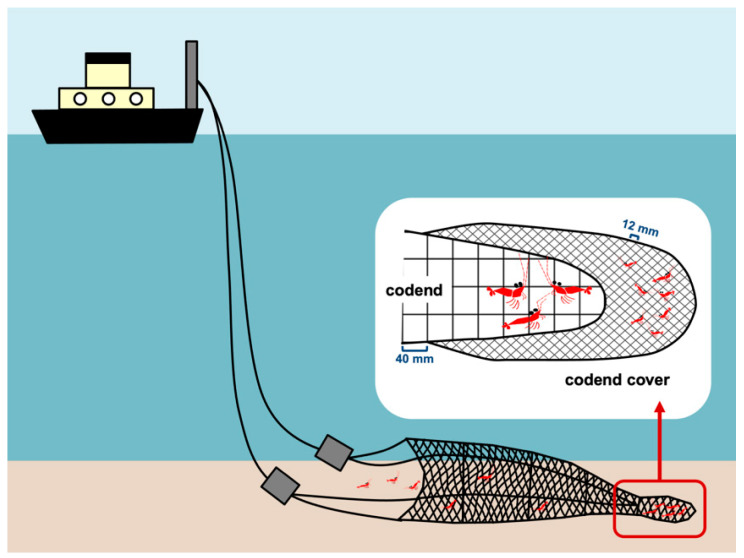
Sampling scheme to collect large and small specimens of *A. antennatus*.

Planella et al. [10] delineated the mating structure of *A. antennatus* in the fishing ground of Palamós Canyon (hereafter “Palamós fishing ground”), Spain, in the summer of 2015. In the present study, *A. antennatus* males were captured on board the same trawling vessel (Nova Gasela) at Palamós fishing ground (500 m depth, 41°54′04′′ N, 3°16′08′′ E) during March (winter) and July (summer) of 2016 to determine the growth and recruitment of males in the area. The winter sampling period (3 March) corresponded to the period when peak numbers of juveniles are detected in the coastal areas of the western Mediterranean [26]. The summer sampling period (7 July) corresponded to the peak of the spawning season, when almost all captured females have at least one spermatophore adhering to the thelycum [10]. The codend had square meshed windows with an aperture of 40 mm (minimum commercial mesh size allowed at the time of this study), and was surrounded by a smaller meshed cover (hereafter “codend cover”), with an aperture of 12 mm usually intended for smaller individuals. Thus, the codend cover captures organisms escaping from the codend (Figure 1). The codend cover was directly attached to the funnel end of the net which was 1.5 m wider and longer than the codend to maintain a good flow of water and avoid masking the codend mesh (see [3]). The codend in this study is commonly used for commercial purposes in the Palamós fishing ground, whereas the codend cover was legally authorised for this scientific sampling procedure. Winter and summer samplings were conducted in the same way. A single haul with the same fishing gear was conducted per sampling period (winter and summer). The towing speed was around two knots and the effective towing duration was two hours. Specimens from the codend and codend cover were independently sexed on board, and a random sample of around 100 males of each group (codend and codend cover) were kept for this study. Each male was classified as juvenile or adult based on the criteria established by Demestre and Fortuño [16] and Sardà and Demestre [18]. All males captured in the codend during March had a fused petasma, and were considered adults. In total, 101 individuals were retained for genotype analyses. Despite some specimens in the codend cover having adult petasma morphology (fused hemipetasmas), most males had two separate hemipetasmas, and were considered as juveniles. In total, 105 of these juveniles were retained for the analysis. In July, all males collected in the codend were adults, and 100 were retained for the analysis. In contrast to the winter collection, all males (except one) captured in the codend cover had fused petasma, and were not considered as juveniles. Ninety-eight of these males were genotyped. All captured specimens were stored on ice on board, and were quickly transported to our laboratory on landing. At the laboratory CL was measured using a digital vernier calliper. A piece of muscle tissue was preserved in 95% ethanol and stored at room temperature until subsequent DNA extraction.

### 2.2. DNA Extraction and Microsatellite Loci Genotyping

From each specimen, DNA was extracted from the muscle tissue using the adjusted phenol-chloroform method proposed by Fernández et al. [28]. Genetic diversity was analysed at 10 microsatellite loci previously described for *A. antennatus* (Aa123, Aa138, Aa1444, Aa667, Aa681, Aa751, Aa956, Aa1061, Aa1195, and Aa818) [29], and amplified with three multiplex PCRs [10]. Resulting amplicons were analysed in an ABI PRISM 3730xl DNA analyser (Applied Biosystems, Foster City, CA, USA) at the Sequencing Unit of the University of Santiago de Compostela (Campus Lugo, Lugo, Spain), and were genotyped using GeneMapper software version 4.0 with GeneScan 500LIZ dye Size Standard (Applied Biosystems) as the internal standard. Genotype data will be available on request.

### 2.3. Statistical and Genetic Analysis

One-way analysis of variance (one-way ANOVA) included in the IBM SPSS statistics version 25 package (Armonk, NY, USA) was used to compare average CL among the four groups of males: (i) adult males captured during winter, (ii) juvenile males captured during winter, (iii) adult males captured in the codend during summer, and (iv) males captured in the codend cover during summer. Subsequently, a post-hoc Scheffe test implemented in the same software was used to identify groups that differed significantly.

Levels of genetic diversity for each of the four male groups were estimated at the 10 loci based on the number of alleles per locus (*N_A_*), allelic richness (*A_R_*), and observed (*H_O_*) and expected (*H_E_*) heterozygosities. Calculations were performed using FSTAT 2.9.3 [30]. Conformance of the observed genotype distributions to their expectations under Hardy–Weinberg equilibrium (HWE) was tested for each sample using the exact probability test of Guo and Thompson [31], which was included in the GENEPOP 4.7.0 software [32]. Deviations from HWE genotype proportions were summarised using the inbreeding coefficient *F_IS_* [33]. Significance levels were adjusted using Bonferroni correction. Null alleles are commonly observed in decapods [29,34], including the *A. antennatus* loci used in this study [35]. For each locus and each male group, null allele frequency was estimated using the Brookfield 1 equation [36], as implemented in Micro-Checker 2.2.3 software [37]. Genetic distinctions between male groups were evaluated by pairwise *F_ST_* estimates using the Weir and Cockerham method [33] in GENEPOP 4.7.0 and their significance from contingency tables of male-group x allele as implemented in the genic differentiation option in GENEPOP 4.7.0 software.

To study the parental contribution of the 2015 local spawners reported by Planella et al. [10] to the male groups analysed here, we simulated several independent F1 offsprings using HYBRIDLAB 1.0 program [38] from the genotypes of these 2015 adults. We first generated 10 independent simulated sets of paired baselines. Each set included a baseline of 100 specimens from the admixture of the adult females (average CL = 38.27 ± 3.76 mm) captured in 2015 and the spermatophores on their thelycum (hereafter “F × S F1”) and another simulated offspring baseline of 100 specimens from females and males (average CL = 22.78 ± 2.59 mm) that were simultaneously caught in 2015 (hereafter “F × M F1”). We used these paired sets of F × M F1 baselines because of the genetic distinction observed between these males and the female spermatophores [10]. Then, we assigned 25 sets of 100 specimens of F × S F1 obtained from independent HYBRIDLAB simulations to each of the 10 baselines sets to obtain 250 replicates (25 × 10) to estimate the accuracy of the assignment of the expected offspring from putative local mates (F × S) in 2015. The size of each set (100 simulated individuals) was comparable to the roughly 100 males in each of the four samples collected in 2016. The Bayesian Rannala and Mountain method [39], as implemented in GENECLASS 2 [40], was used in all performed assignments. Individuals with an assignment probability to each of the two baselines <0.01 were considered as having another source. Similarly, we assigned 25 sets of F × M F1 simulated offspring to the 10 sets of baselines to estimate the accuracy of assignment of the expected offspring from local adults (F × M) caught during the 2015 spawning season. Finally, we assigned our 2016 samples (winter codend cover, winter codend, summer codend cover, and summer codend) to each of the 10 baseline sets to compare outcomes with those obtained above for the expected offspring from local 2015 shrimps. These comparisons were done using ANOVA and post-hoc Scheffe test as indicated for CL analysis.

Assignment tests were also used to determine the relationships between the males collected in summer versus winter. This approach was addressed to detect the most likely origin of summer males using the winter males as a baseline. Two additional sets of samples of adult male *A. antennatus* captured during the winter of 2016 at nearby grounds were incorporated as a potential source of migratory specimens. One set was from Cap de Creus Canyon (Roses fishing ground, 42°21′17′′ N, 3°24′22′′ E) (*n* = 55), and the other set was from Blanes Canyon (Blanes fishing ground, 41°35′85′′ N, 2°50′56′′ E) (*n* = 54), which were situated at approximately 60 km northward and southward of the Palamós fishing ground, respectively. Exact probability tests were used to compare the assignment pattern of the summer collections to the results obtained from the Palamós winter baselines.

## 3. Results

### 3.1. Comparison of Carapace Length among Male Groups

**Figure 2:**
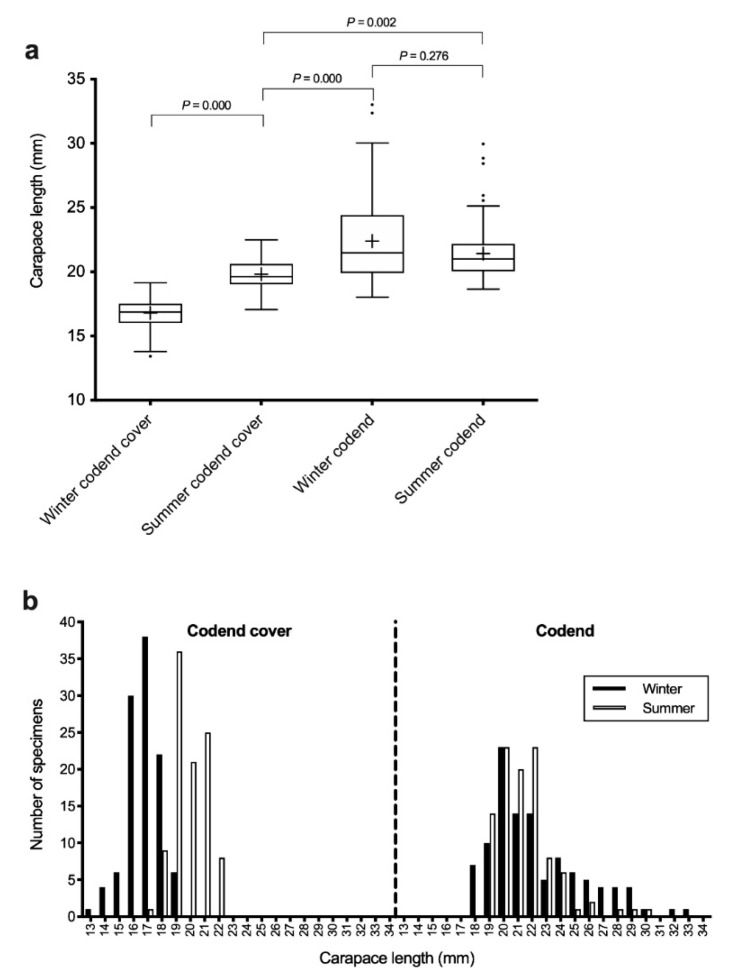
Carapace length (CL) of male *Aristeus antennatus* groups captured at Palamós fishing ground, Spain. (**a**) Box and whisker plot, the median is shown by the line that cuts through the box, the mean is represented by a cross, *P* values were obtained from Post-hoc Scheffe test; (**b**) Size-frequency distributions.

The *A. antennatus* juveniles captured in the codend cover during winter had the lowest mean CL (16.79 ± 1.15 mm), which significantly differed to all other analysed groups (Figure 2). Males captured in the codend cover during summer were significantly larger than winter juveniles, with a mean CL of 19.82 ± 1.10 mm; however, these males were smaller than adults collected in the codend during winter or summer. Adult males captured in the codend during summer had a mean CL of 21.43 ± 2.06 mm, which was not significantly different to the mean size of winter adults (22.39 ± 3.34 mm). The largest adult male specimens were among those captured in winter.

### 3.2. Genetic Diversity

The 10 loci were polymorphic in all analysed male groups, with the number of alleles per locus (*N_A_*) ranging from three (locus Aa751 in all groups) to 25 (locus Aa138 in the summer codend group) (Table 1). The mean allelic richness (*A_R_*) ranged from 8.8 (summer codend cover group) to 9.7 (summer codend group). The average observed heterozygosity (*H_O_*) among loci in the male groups reached 0.495 in winter juveniles to 0.517 in summer adults from the codend, and was consistently below the average expected heterozygosity (*H_E_*). After Bonferroni correction, analysis of locus by locus deviations from genotype HWE expectations showed significant departures in five loci of the winter codend cover group (Aa1444, Aa667, Aa751, Aa1061, and Aa818), six loci of the winter codend group (Aa1444, Aa667, Aa681, Aa751, Aa1061, and Aa818), six loci in the summer codend cover group (Aa123, Aa1444, Aa667, Aa681, Aa1061, and Aa818), and five loci in summer codend group (Aa1444, Aa681, Aa751, Aa1061, and Aa818). Positive *F_IS_* indicated that a heterozygote deficit was obtained in all groups. However, after Bonferroni correction, only males captured in the codend cover during summer showed an overall significant departure from HWE. A maximum null allele frequency of 0.195 was estimated by Micro-Checker software at locus Aa818 in the summer codend cover male group.

### 3.3. Genetic Divergence

Pairwise *F_ST_* values were low among all male groups (range: 0.0000–0.0010) (Table 2). After Bonferroni correction, significant differences were documented between juvenile and adult males collected during winter (*F_ST_* = 0.0010, *P* = 0.0004), and also between winter and summer groups in the codend cover (*F_ST_* = 0.0008, *P* = 0.0003).

**Figure 3:**
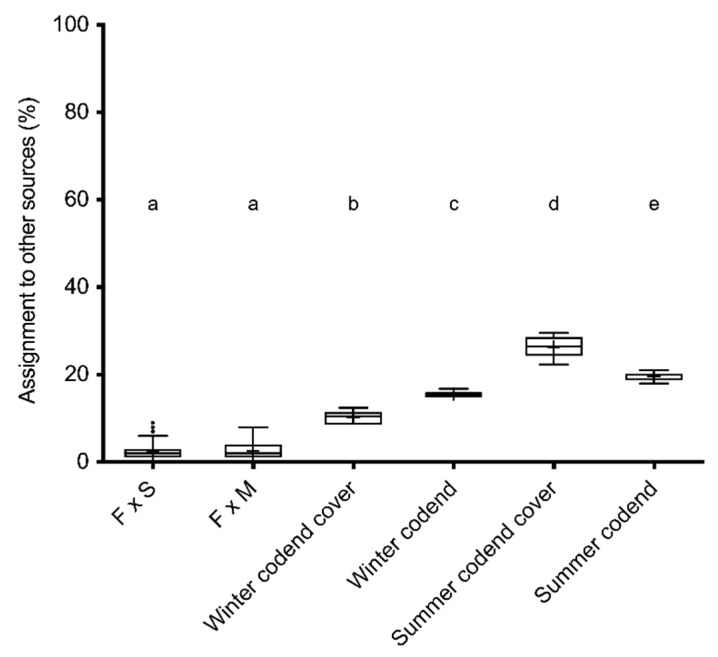
Distribution of the proportion (%) of assignment to other sources of the simulated offspring sets (F × S and F × M, see text) and the 2016 samples. Letters (a, b, c, d and e) indicate groups identified by post-hoc Scheffe test.

A limited accuracy of the baselines was indicated in the assignment of the simulated datasets (Table 3). In particular, a large portion of simulated F × M F1 individuals were assigned to F × S F1 baselines, likely reflecting the genetic similarity between the males and spermatophores sampled in 2015 and a limited contribution of the males to the spermatophores reported by Planella et al. [10]. Nevertheless, the assignment of all groups of males captured in 2016 significantly differed from simulated individuals. In all cases, the largest assignment was at the F × S F1 baseline. In addition, all male groups in 2016 significantly incorporated specimens from other sources (from 10.19 to 26.33%, Table 3), when compared to the reference simulated F1 individuals from local spawners. The largest assignment to other sources was detected for small sized males collected in the codend cover during the 2016 summer. The assignment pattern of this group also differed to that of winter juveniles (Figure 3).

The geographical assignment of captured males (Table 4) showed that the winter codend and codend cover group from the Palamós fishing ground were weakly assigned to the origin (67.3 and 43.8%, respectively). The assignment of these two groups confirmed that recruitment from other grounds occurs. This recruitment was mostly from Blanes Canyon, based on juveniles from the codend cover group and adults from the codend group. The assignment pattern of the two summer groups statistically differed to that recorded for the winter group (*P* < 0.01 in all comparisons). The summer codend and codend cover groups were more closely correlated to winter adults collected in the codend compared to winter juveniles collected in the codend cover. Only 18.4% of summer codend cover males were assigned to winter juveniles, with this percentage being slightly lower in the summer codend specimens (13.0%). In fact, the two summer groups had similar assignment patterns (exact test *P* = 0.1686), with more than 40% of specimens originating from outside of the Palamós ground.

## 4. Discussion

### 4.1. Growth and Recruitment of A. antennatus in Submarine Canyons

The males captured at a depth of 500 m in the codend cover during winter were classified as juveniles because they lacked fused petasma. The average CL (16.79 mm) of these males supports observations of juvenile sizes from other Mediterranean grounds [8,11,24]. The winter juvenile group from Palamós was composed of smaller specimens compared to the adult males captured in the codend. This latter group had an average CL of 22.39 mm, with this size exceeding that of males at first maturity (20.81 mm) reported by Carbonell et al. [14]. In fact, the average CL of the winter adult male group captured in the codend was similar to that of adult males captured during the 2015 spawning season (22.78 mm). Thus, these winter adults could be related to, or be the same as, the adult male group analysed by Planella et al. [10], as no significant genetic differentiation (*F_ST_* = −0.00022, *P* = 0.4899) was recorded between these two male groups. Greater genetic divergence of these winter males from males that released spermatophores into the female thelycum during the spawning season was recorded (*F_ST_* = 0.00318, *P* = 0.0812). However, analysis of the parental contribution of 2015 summer local spawners based on simulated offspring showed that the greatest assignment was to F × S F1 out of all male groups captured in 2016. The estimated contribution of F × M F1 was lower (Table 3). Therefore, the winter adult codend group might have included both adult males related to the 2015 summer local males and offspring that hatched early during the spawning season. These latter individuals had more time to grow compared to specimens captured in the codend cover. The admixture of these two groups also might explain the large variance observed in CL in this sample.

The males captured in the codend cover during summer were larger compared to those captured during winter. Despite these summer codend cover males having adult morphology (fused petasma), their average size (CL = 19.82 mm) was lower than that reported for first maturity. Certainly, this group could be composed of winter juveniles that had grown since the last spring. A growth rate of 2 mm per month was estimated for juvenile *A. antennatus* cohorts in June–July at the fishing grounds of the Catalan Sea [41]. Sardà and Company showed that juveniles of <16 mm CL were abundant in the bottom sea areas of submarine canyons, where they remained for 6–9 months [8]. Later, the ontogenic migration of pre-adults to depths of less than 900 m occurred, with these individuals becoming incorporated into the *A. antennatus* fishery at 400–800 m depth. Despite already showing the adult petasma fused phenotype, pre-adult shrimps were considered to be 20–28 mm CL. Thus, all males (except a few individuals >28 mm CL) captured during summer in the current study could be pre-adult specimens, despite the mean CL (21.43 mm) of those captured in the codend exceeding that described for males at first maturity. Sex determination in *A. antennatus* is not known; however, a ZZ/ZW sex chromosome mechanism has been documented in Penaeid shrimps [42]. This mechanism should produce a balanced sex ratio (1:1) in offspring. In blue and red shrimp, balanced (1:1) sex ratios in fishing grounds are only detected in autumn and winter, after the spawning season. In contrast, during the spawning season, and especially in summer, there is strong female bias both in biomass and numbers [5,11], suggesting a spatial sexual segregation at that time. Planella et al. [10] observed that males captured at fishing grounds during the spawning season had a limited contribution to the spermatophores attached to the thelycum of females. Unlike females, *A. antennatus* males have a clear size limit, being small-sized throughout their entire adult life, which complicates attempts to distinguish the cohorts of older specimens [43]. However, our genetic analyses correlated most males captured in summer to the offspring of 2015 spawners (Table 3). Therefore, the upward migration to shallower waters in summer mostly involves females of all sizes and pre-adult males.

### 4.2. Geographical Origin of Males Recruited into the Fishery

The blue and red shrimp is a deep-sea benthic crustacean that has a widespread dispersal potential [44], contributing to high gene flow and low levels of spatial genetic heterogeneity across the fishing grounds of the Mediterranean Sea [6,34,35,45,46,47,48,49]. The horizontal displacement of *A. antennatus* might occur by passive and active dispersal mechanisms. Orsi-Relini et al. [50] suggested that, after the major passive horizontal displacement of *A. antennatus* larvae by surface currents, juveniles and adults might initiate an active, but slow, return migration against weak deep-sea currents. Scant evidence has been reported on adult migration, with just a single tagging study in the Ionian Sea (Mediterranean). This study showed that most recaptured specimens (21 out of 693 tagged shrimps) were collected in the same area shortly after release; however, two specimens were recaptured at one and nine months after release in deeper areas several nautical miles (6 to 10 nautical miles) from the release site [51].

*Aristeus antennatus* eggs and larvae do, however, disperse passively, based on their presence in the upper water layers [20,23]. In our study region, the general oceanic circulation is part of a cyclonic circuit called the Northern Current (NC). This is a well-defined western Mediterranean current that extends to 300–400 m depth, with a general southwest flow that follows the continental slope from Italy to Spain [52,53]. Using hydrodynamic models to predict connectivity among *A. antennatus* populations by passive egg and larval drift among submarine canyons in our study region, Clavel-Henry et al. predicted a global pattern of southward dispersal, according to the Northern Current [22]; however, the authors also predicted high average retention rates in submarine canyons in some models (reaching up to 60%). Our genetic analyses support these predictions, showing assignation rates of 50–70% for all juveniles and pre-adult males captured in 2016 at the Palamós fishing ground to the simulated F1 between females and their spermatophores sampled in 2015 (Table 3), as well as the contribution of migrants from northern grounds (Table 4).

The limited contribution of winter juveniles to the summer pre-adult groups indicates that juvenile dispersal is a continuous process through the year that contributes to recruitment at the fishing grounds. The Levantine Intermediate Water (LIW) and the Western Mediterranean Deep Water (WMDW) flow below the NC and in the same direction [54]. The abundance of adult females appeared to be correlated to LIW; however, *A. antennatus* juveniles were more abundant in the fishing grounds of the study region when WMDW was present [15,55]. Anticyclonic eddies that extend their effects to deeper waters produce inversions of the general southwest flow, and recur along the coasts of the northwest Mediterranean [53]. Such eddies are singularly frequent in the Blanes Canyon area [56]. Thus, an anticyclonic eddy in the Blanes Canyon area might also explain the higher estimated contribution of *A. antennatus* specimens from this region to the Palamós fishing ground, rather than the Roses Canyon.

Larval and juvenile migration from other grounds, rather than null alleles, could explain the observed departures from Hardy–Weinberg genotypic expectations in our groups [57]. This phenomenon contributed towards maintaining high local diversity and weak genetic divergence at a regional scale, similar to that observed in *A. antennatus* fishing grounds elsewhere [34,35]. Certainly, very little is known about how deep-sea oceanographic processes influence demersal fisheries. Despite this, Puig et al. [58] highlighted the importance of considering how the environment and species interact to exploit deep-water resources sustainably. The features documented in the current study could be applied by fisheries for other deep-sea crustacean and fish species. In particular, how the juveniles of these various groups disperse among and replenish regional grounds should be incorporated in the management of potential targets by future regional demersal fisheries [54]. Currently, output management measures (quotas) are not implemented in the Mediterranean demersal fisheries and ongoing programs involving reductions in fishing time are insufficient to restore and maintain fish stocks below fishing mortality levels capable of producing maximum sustainable yields [4].

## 5. Conclusions

This study provides new evidence on the growth, recruitment, and geographical origin of male *A. antennatus* in fishing grounds. This information could be applied to facilitate the sustainable exploitation of this marine resource. Our results showed that males frequenting the fishing ground were mostly recruited from local spawners, but with contributions from other sources. Our findings indicate: (i) upward summer vertical movement towards the fishing grounds of pre-adult males that hatched the preceding year, and (ii) horizontal displacement of juvenile males from adjacent fishing grounds by deep currents, complementing larval dispersal through surface waters that occurs shortly after spawning. However, it was not possible to resolve which males were recruited for spawning and made a major contribution to spermatophores. Certainly, a small number of large and mature males are captured with females during the spawning season (Figure 2a and [5]). However, male-biased sex ratios of 2:1 are detected in summer at depths below 1000 m, in unexploited regions [7,11], with such individuals being in the best biological condition for mating and spawning [24].

## Figures and Tables

**Table 1:** Genetic diversity within male *Aristeus antennatus* groups captured at Palamós fishing ground, Spain.

Cohort		Aa123	Aa138	Aa1444	Aa667	Aa681	Aa751	Aa956	Aa1061	Aa1195	Aa818	Average
Winter codend cover	*N_A_*	4	19	13	9	20	3	6	8	4	4	9.0
	*A_R_*	3.913	18.777	12.752	8.842	19.567	3.000	5.988	7.884	3.895	4.000	8.862
	*H_O_*	0.485	0.829	0.490	0.392	0.637	0.144	0.571	0.429	0.591	0.352	0.495
	*H_E_*	0.614	0.880	0.754	0.702	0.795	0.302	0.666	0.682	0.608	0.582	0.660
	*F_IS_*	0.210	0.059	0.350 *	0.442 *	0.199	0.521 *	0.142	0.371 *	0.028	0.394 *	0.251
	*Nu*	0.091	0.043	0.155	0.181	0.071	0.122	0.054	0.173		0.147	
Winter codend	*N_A_*	5	20	12	7	22	3	6	8	5	6	9.4
	*A_R_*	4.930	19.904	11.937	6.918	21.436	3.000	6.000	7.792	4.991	5.995	9.290
	*H_O_*	0.485	0.812	0.490	0.449	0.545	0.126	0.644	0.475	0.654	0.356	0.506
	*H_E_*	0.610	0.896	0.715	0.699	0.856	0.271	0.665	0.697	0.596	0.625	0.665
	*F_IS_*	0.205	0.094	0.316 *	0.358 *	0.364 *	0.534 *	0.032	0.318 *	−0.097	0.430 *	0.239
	*Nu*	0.075	0.048	0.135	0.152	0.186	0.128		0.129		0.153	
Summer codend cover	*N_A_*	5	19	13	6	19	3	6	6	5	6	8.8
	*A_R_*	4.959	18.915	12.937	5.979	18.875	3.000	5.999	6.000	4.918	5.998	8.758
	*H_O_*	0.418	0.837	0.500	0.479	0.541	0.188	0.561	0.459	0.622	0.347	0.496
	*H_E_*	0.625	0.911	0.762	0.694	0.828	0.287	0.682	0.696	0.628	0.689	0.681
	*F_IS_*	0.330 *	0.082	0.344 *	0.310 *	0.347 *	0.347	0.177	0.340 *	0.008	0.497 *	0.272 *
	*Nu*	0.116	0.040	0.152	0.131	0.166	0.080	0.057	0.136		0.195	
Summer codend	*N_A_*	6	25	12	7	20	3	6	7	6	6	9.8
	*A_R_*	5.933	24.513	11.955	6.958	19.806	3.000	6.000	6.998	5.877	5.999	9.704
	*H_O_*	0.520	0.800	0.531	0.542	0.640	0.096	0.610	0.485	0.610	0.306	0.517
	*H_E_*	0.647	0.910	0.735	0.690	0.809	0.281	0.684	0.747	0.612	0.618	0.675
	*F_IS_*	0.196	0.120	0.278 *	0.215	0.209 *	0.660 *	0.108	0.351 *	0.004	0.504 *	0.235
	*Nu*	0.069	0.060	0.120	0.083	0.091	0.155		0.151		0.185	

*N_A_*, Number of alleles; *A_R_*, allelic richness; *H_O_*, observed heterozygosity; *H_E_*, expected heterozygosity; *F_IS_*, inbreeding coefficient; *Nu*, null allele frequency; *, Significant departure from Hardy–Weinberg equilibrium after Bonferroni correction.

**Table 2:** Genetic divergence (*F_ST_*) between male *Aristeus antennatus* groups.

	Winter Codend Cover	Summer Codend Cover	Winter Codend
Summer codend cover	0.0008 ^1^		
Winter codend	0.0010 ^1^	0.0000	
Summer codend	0.0004	0.0004	0.0000

^1^ Significant *F_ST_* values after Bonferroni correction.

**Table 3:** Assignment distribution (%) of male *Aristeus antennatus* groups to simulated offspring from 2015 spawner genotypes (see text).

	Replicates	F × S F1 Baseline ^1^	F × M F1 Baseline ^1^	Other Sources
F × S F1	250	69.7 (16.3)	28.1 (15.9)	2.2 (1.8)
F × M F1	250	47.2 (16.9)	50.2 (16.8)	2.6 (1.8)
Winter codend cover	10	56.6 (16.4)	33.2 (15.7)	10.2 (1.3)
Summer codend cover	10	44.4 (13.9)	29.3 (13.4)	26.3 (2.2)
Winter codend	10	54.5 (13.3)	30.0 (13.3)	15.5 (0.6)
Summer codend	10	49.10 (12.5)	31.3 (12.3)	19.6 (1.0)

^1^ F, females; S, spermatophores; M, males.

**Table 4:** Assignment results (%) of male *Aristeus antennatus* groups captured during the summer at Palamós fishing ground, Spain.

	Baselines				
	Palamós Winter Codend	Palamós Winter Codend Cover	Roses Winter Codend	Blanes Winter Codend	Other Sources
Palamós winter codend	67.3	7.9	4.0	13.9	6.9
Palamós winter codend cover	21.9	43.8	10.5	20.0	3.8
Palamós summer codend	41.0	13.0	14.0	25.0	7.0
Palamós summer codend cover	39.8	18.4	5.1	24.5	12.2

## Data Availability

The data presented in this study are available from the corresponding author on reasonable request.
